# Next-Generation Sequencing of the Porcine Skeletal Muscle Transcriptome for Computational Prediction of MicroRNA Gene Targets

**DOI:** 10.1371/journal.pone.0042039

**Published:** 2012-07-27

**Authors:** Tara G. McDaneld, Tim P. L. Smith, Gregory P. Harhay, Ralph T. Wiedmann

**Affiliations:** 1 Genetics and Breeding Research Unit, United States Department of Agriculture/Agricultural Research Service/USDA/Meat Animal Research Center, Clay Center, Nebraska, United States of America; 2 Animal Health Research Unit, United States Department of Agriculture/Agricultural Research Service/USDA/Meat Animal Research Center, Clay Center, Nebraska, United States of America; 3 Reproductive Research Unit, United States Department of Agriculture/Agricultural Research Service/USDA/Meat Animal Research Center, Clay Center, Nebraska, United States of America; Auburn University, United States of America

## Abstract

**Background:**

MicroRNA are a class of small RNAs that regulate gene expression by inhibiting translation of protein encoding transcripts through targeting of a microRNA-protein complex by base-pairing of the microRNA sequence to cognate recognition sequences in the 3′ untranslated region (UTR) of the mRNA. Target identification for a given microRNA sequence is generally accomplished by informatics analysis of predicted mRNA sequences present in the genome or in databases of transcript sequence for the tissue of interest. However, gene models for porcine skeletal muscle transcripts in current databases, specifically complete sequence of the 3′ UTR, are inadequate for this exercise.

**Methodology/Principal Findings:**

To provide data necessary to identify gene targets for microRNA in porcine skeletal muscle, normalized cDNA libraries were sequenced using Roche 454 GS-FLX pyrosequencing and *de novo* assembly of transcripts enriched in the 3′ UTR was performed using the MIRA sequence assembly program. Over 725 million bases of sequence were generated, which assembled into 18,202 contigs. Sequence reads were mapped to a 3′ UTR database containing porcine sequences. The 3′ UTR that mapped to the database were examined to predict targets for previously identified microRNA that had been separately sequenced from the same porcine muscle sample used to generate the cDNA libraries. For genes with microRNA-targeted 3′ UTR, KEGG pathways were computationally determined in order to identify potential functional effects of these microRNA-targeted transcripts.

**Conclusions:**

Through next-generation sequencing of transcripts expressed in skeletal muscle, mapping reads to a 3′ UTR database, and prediction of microRNA target sites in the 3′ UTR, our results identified genes expressed in porcine skeletal muscle and predicted the microRNA that target these genes. Additionally, identification of pathways regulated by these microRNA-targeted genes provides us with a set of genes that can be further evaluated for their potential role in skeletal muscle development and growth.

## Introduction

Since their recent discovery, small RNAs have demonstrated a role in multiple developmental processes. MicroRNA (miRNA) are a class of small RNA, approximately 18–26 nucleotide long, that are encoded by nuclear genes that produce characteristic stem-loop RNA structures when transcribed. During processing from the primary transcript, the mature miRNA sequence is loaded into an RNA:protein complex known as the “RNA induced silencing complex” [RISC: **1, 2**]. The sequence of the miRNA loaded in the complex targets the RISC to specific binding sites in the 3′ untranslated region (UTR) of mRNA transcripts, resulting in decreased translation of the targeted gene product [**2]**, [3]. MicroRNA dictate mRNA targeting and translation based on the number and type of base-pair matches in the 3′ UTR and binding site of the miRNA called the seed sequence [**4]**–[6]. As a result of decreased translation of their cognate targets, miRNA have been reported to guide developmental decisions including cell fate, cell cycle progression, apoptosis, adipocyte differentiation, and processes that alter muscle development and growth including myoblast proliferation, differentiation, and skeletal muscle hypertrophy [**6]**–[14].

With the rapid identification of miRNA sequences across multiple species, current research has begun to focused on evaluation of the genes for which the miRNA target and on prediction of biological function. We and other labs have previously identified porcine miRNA expressed in skeletal muscle at different stages of development [**15]**–[19]. Evaluation of the transcriptome including the 3′ UTR is essential to successfully identify all miRNA gene targets, although the current genomic models for porcine are inadequate for this process. The current version of the RefSeq (reference sequence) database at NCBI contains only 20,578 *Sus scrofa* mRNAs, most of which are computationally predicted rather than experimentally validated, and include multiple splice forms. Therefore, to evaluate further the role of miRNA in skeletal muscle development, the objectives of the current experiment were to 1) reconstruct the porcine skeletal muscle transcriptome and 2) computationally identify miRNA-targeted genes in the transcriptome and biological pathways by which these genes mediate skeletal muscle development and growth.

## Results and Discussion

### 454 Sequencing of cDNA Libraries

To identify the transcripts expressed in porcine skeletal muscle and evaluate the 3′ UTR of these transcripts for miRNA binding sites, two cDNA libraries were constructed from porcine skeletal muscle RNA, normalized to remove highly abundant transcripts, and subjected to next-generation pyrosequencing producing 2,324,269 reads. The first library (1,368,147 reads; 400 Mb of sequence) was constructed using a poly-T primer for reverse transcription that contained a GsuI restriction enzyme recognition site to simultaneously target the 3′ UTR, which contains the miRNA binding site, and remove the poly-A tail from the amplified transcripts since homopolymers can result in errors during 454 pyrosequencing. A second cDNA library (956,903 reads; 326 Mb of sequence) was constructed using random hexomers to prime cDNA synthesis and generate sequences that would cross any GsuI restriction sites present within the body of the transcript and produce full-length gene models.

### Assembly and Annotation of Transcripts

To reconstruct the transcripts present in skeletal muscle, all sequence reads (2,324,269) from 454 pyrosequencing were assembled with the MIRA genome sequence assembler [**20**]. Initially, all reads from the random hexomer cDNA library were assembled into contigs to which reads from the GsuI cDNA library were incorporated to increase depth of sequence reads across contigs for the expressed transcripts and increase sequence reads for the underrepresented 3′ UTR region of the transcripts. Initial results from the pyrosequencing and MIRA assembly are presented in Table 1 for 1,977,184 reads that assembled into 18,202 contigs after removing contigs comprised of <10 reads (contig set identified as MINREADS). The MINREADS contigs were compared to the *Sus scrofa* RefSeq RNA database, which contains 20,578 unique accessions corresponding to 19,077 unique genes, using BLASTN (basic local alignment search tool for nucleotides) to determine if our current data set of sequence reads was representative of the transcripts expressed in skeletal muscle.

**Table 1:** Results for pyrosequencing, MIRA assembly, and comparison to RefSeq mRNA and protein databases.

Pyrosequencing results	
Number of total bases	725,184,671
Number of total sequence reads	2,324,269
**MIRA assembly**	
Number of reads assembled	1,616,646
Number of contigs	121,071
Average size of contigs	635
Largest contig	48,138
**RefSeq Results**	
Number of contigs evaluated	18,202
Contigs (>10 reads, 90% alignment) that hit to RefSeq mRNA (BLASTN)	5,688
Individual genes hit by contigs	4,541
Contigs (> 10 reads, 90% alignment) that hit to RefSeq protein (BLASTX)	2,243
Individual proteins hit by contigs	1,416

There were 12,096 MINREADS contigs (66.5%; Table 1) that displayed matches to 8,536 different RefSeq transcripts (average 1.4 contigs per transcript). Upon evaluation of the BLASTN results, spurious matches were identified that resulted from segments of contigs containing repetitive sequence found across multiple RefSeq transcripts. To reduce this artifact, BLASTN results were filtered by adding a requirement that the span of the alignment between contig and RefSeq transcript model be a minimum of 90% of the entire length of the contig. This filter is conservative in that some of the contigs that contain extensive 3′ UTR sequence that is not present in the RefSeq gene models will be removed from the dataset despite a sufficient alignment that they could be confidently assigned to a RefSeq accession. The filter reduced the number of contigs aligning to annotated transcripts to 5,688 contigs that matched 4,541 unique RefSeq accessions, permitting 31% of the total MINREADS contigs to be assigned transcript annotation (Table 1). This filter set indicates that our sequencing results of the transcripts expressed in porcine muscle represent >22% of *Sus scrofa* RefSeq annotated transcripts.

The MINREADS contigs were also translated and compared to the RefSeq protein database, which contains 24,846 unique accession numbers, using BLASTX (basic local alignment search tool for proteins). There were 1,416 MINREADS contigs that matched to 2,234 proteins (Table 1) in the RefSeq protein database when the data was filtered to remove contigs that comprised of <10 reads as described above. When the contigs were filtered to only include contigs that aligned to >90% of the protein, 5,496 MINREADS contigs matched to 4,788 proteins (Table 1) suggesting that less than 20% of the known proteins in the RefSeq protein database are present in our data set of transcripts expressed in porcine skeletal muscle. A subset of contigs that matched to the RefSeq RNA and protein databases were evaluated for open reading frames (ORF) using Geneious [**21**] to determine if the coding sequence (CDS) that matched to the RefSeq database was within an ORF. All of the contigs evaluated had an ORF that matched to the CDS, suggesting that through 454 pyrosequencing we were able to assemble full-length transcripts.

The 18,202 MINREADS contigs were also compared to the draft porcine genome (Build 10) using BLAT to identify potential genes not present in the RefSeq database. There were 17,559 contigs (96.5%) that aligned to the genome, which was reduced to 14,268 (78.4%) if a requirement for alignment of >90% of the length of the contig was added. Additionally, approximately 33% of the MINREADS contigs did not show any match to the current mRNA database, which increased to 69% if the requirement for 90% length-of-contig match was added. Further comparison against the entire nucleotide database, determined that these contigs matched either expressed sequence tags (ESTs) previously derived from porcine cDNA libraries, porcine DNA sequence, human DNA sequence or BAC (bacterial artificial chromosome) clones not present in the current RefSeq databases. For the contigs that hit to ESTs, >95% corresponded to the 3′ UTR for the gene represented by the EST sequence. The abundance of sequences for the 3′ UTR is a result of our current data set being enriched for 3′ UTR sequences as the first cDNA library was purposely targeting the region that contains the binding site for miRNA. Additionally, there were a number of contigs that did not match porcine sequence in the current database, yet matched to human DNA and BAC sequence. These data suggest that these contigs represent regions of the porcine genome that have not been fully annotated.

### Identification of Full Length 3′ UTR

The second goal of the current study was to computationally identify miRNA that target transcripts expressed in skeletal muscle of porcine. However, upon analysis of the contigs created, we discovered that a large portion of the transcripts (>27%) did not map reliably to the current porcine genome. As a result, we were not able to properly identify the 3′ UTR and miRNA that target these transcripts. Subsequently, for this study we focused on the 3′ UTR expressed in porcine skeletal muscle by utilizing a 3′ UTR database containing *Sus scrofa* 3′ UTR. Initially, all sequence reads from 454 sequencing of both cDNA libraries described herein were mapped to the *Sus scrofa* 3′ UTR represented in the 3′ UTR database (http://utrdb.ba.itb.cnr.it/) using the Newbler sequence analysis software (Roche 454, Branford, CT). Of the 5,134 full 3′ UTR represented in the database for *Sus scrofa*, 895 3′ UTR had 100% coverage of the mapped sequence reads from porcine skeletal muscle. This subset of 3′ UTR were then computationally evaluated to identify miRNA target sites for miRNA that we have previously identified to be expressed in porcine skeletal muscle (Table S1).

### Identification of MicroRNA Gene Targets and KEGG (Kyoto Encyclopedia of Genes and Genomes) Pathways

We have previously used a digital expression profile approach to evaluate miRNA abundance in porcine skeletal muscle during prenatal and postnatal development [**15**]. For the current experiment, we set out to identify the genes that these miRNA target. To accomplish this, the 33 most abundant miRNA in porcine skeletal muscle (Table S1) were computationally matched to the subset of 895 porcine 3′ UTR that were identified by mapping the sequence reads described herein to a 3′ UTR database (Table S2; Table S3). There are a number of online programs that can be used to computationally identify miRNA gene targets. We used the MiRanda and TargetScan software to evaluate our current subset of 3′ UTR for miRNA target sites. The MiRanda software predicts miRNA gene targets by searching for complementary miRNA sequence in the 3′ end of the target gene sequence [**22]**, [23]. However, the TargetScan software is a more conservative program that predicts miRNA gene target sites by identifying 7mer and 8mer sequences that match the seed sequence for the targeting miRNA [**24]**–[26] **.** Based on the MiRanda analysis (Table S2), we determined that 525 of the 895 3′ UTR (59%) contain at least one miRNA targeting site and 81% of these 3′ UTR were targeted by more than one miRNA that we have previously identified to be expressed in porcine skeletal muscle (Table S1). For genes targeted by more than one miRNA, this multiplicity can be the result of different miRNA sharing the same target sequence or the transcript having multiple target sites for a number of miRNA. In comparison, the TargetScan analysis program identified that 817 of the 895 3′ UTR (91%) contain at least one miRNA targeting site for miRNA that we have previously identified to be expressed in porcine skeletal muscle (Table S3). Of the 3′UTR that were identified to be targeted by MiRanda (525 3′ UTR) and TargetScan (817 3′ UTR), there were 178 that demonstrated overlap between the two analysis programs (Table S4). Additionally, variation between the specific miRNA that target each of these 3′ UTR existed between the MiRanda and TargetScan analysis. This difference between the MiRanda and TargetScan analysis may be due to the different algorithms and databases (Ensembl data or UCSC data) that are used to computationally identify miRNA-targeting sequence for the two analysis programs [**22]**–[26]. This variation in prediction of miRNA targets between prediction programs has been demonstrated previously suggesting that using more than one prediction program for computational identification of miRNA gene targets is of importance to begin to predict genes that are regulated by miRNA targeting.

To examine the function of these transcripts with 3′ UTR targeted by miRNA, KEGG pathways [**27**] were determined using DB visualizer (http://www.dbvis.com; Table S2; Table S3). Pathways predicted for these transcripts encompass an array of biological processes and molecular function including regulation of cellular processes, signaling pathways and structures that regulate skeletal muscle growth. These pathways include protein synthesis, export, absorption, and digestion, which are important in the neonate animal in which skeletal muscle is a fast growing protein mass that exhibits high levels of protein accumulation and increased numbers of nuclei in the muscle fibers [**28]**, [29]. Muscle-specific miRNA (miR-1, miR-133, and miR-206) have also been reported to increase in abundance during muscle cell differentiation and regulate different stages of myogenesis in which protein processing is of great importance [**8]**, [10]–[14].

A number of the predicted miRNA-targeted genes were associated with signaling pathways that impact skeletal muscle growth and function, including calcium ion binding, actin structure, and insulin signaling pathway [**30–32**; Table S2; Table S3; Table S4]. Additionally, several signaling pathways previously reported to have a crucial role in mediation of skeletal muscle development were associated with predicted miRNA-targeted genes including JAK-STAT (janus kinase-signal transducer and activator of transcription), MAPK (mitogen activated protein kinase), mTOR (mammalian target of rapamycin), PPAR (peroxisome proliferator-activated receptor), TGF-b (transforming growth factor-beta), and Wnt signaling pathways [**33]**–[39].

As discussed previously, we and other groups have identified the muscle-specific miRNA miR-1, miR-206, and miR-133 as miRNAs that increase in abundance during differentiation and play a role in mediation of skeletal muscle development and growth [**8]**–[15]. These miRNA has been reported to regulate different stages of myogenesis; miR-133 increases proliferation of C_2_C_12_ myoblasts, whereas miR-206 and miR-1 promote differentiation [**13**]. Approximately 12% of the genes predicted in this study to be targeted by miRNA were predicted as gene targets for both miR-1 and miR-206 (TargetScan uses the same seed sequence for miR-1 and miR-206 and are represented by miR-1 in Table S3). These miRNA have 18 of 22 nucleotide similarity and share the same seed sequence that determines targeting of the miRNA to the transcript. Genes targeted by miR-1, miR-206, and miR-133 function in multiple roles of skeletal muscle structure including the actin cytoskeleton [**40, 41**; Table S2; Table S3]. The actin filament is a major component of the thin filament of skeletal muscle that has a role in muscle structure and contraction [**42]**, [43]. Additionally, other KEGG pathways associated with genes targeted by miR-1, miR-206, and miR-133 implicated in muscle function include calcium ion binding and calcium ion homeostasis, which have a role in skeletal muscle contraction [**44]**, [45].

### Conclusions

Identification of miRNA targets in the 3′ UTR of genes is a vital step in determining the biological functions that miRNA play in developmental processes. To identify miRNA-targeted genes expressed in porcine skeletal muscle, the transcriptome for porcine skeletal muscle was evaluated utilizing next-generation sequencing technology. Through sequencing of the transcriptome and evaluation of a 3′ UTR database, miRNA-targeted genes in our dataset were computationally identified. Overall, data from the current experiment have predicted a set of miRNA-targeted genes that can be evaluated further for regulation of skeletal muscle growth and development. While we predicted a number of miRNA that are highly expressed in skeletal muscle and target 3′ UTR of transcripts expressed in skeletal muscle, there is a large portion of these expressed miRNA (Table S1) that have not been previously reported to regulate skeletal muscle growth. This suggests that further research needs to be completed to expand our understanding of how these miRNA target genes that regulate skeletal muscle growth. Additionally, utilization of this protocol for next-generation sequencing of transcriptomes has resulted in a preliminary gene model for production livestock transcriptomes that can be utilized for future experiments.

## Materials and Methods

### RNA Extraction and Construction of Transcriptome Libraries

Total RNA was isolated from adult porcine *biceps femoris* using TRI-reagent (Invitrogen, Carlsbad, CA) following manufacturers instructions. The mRNA fraction was isolated using a Dynabead mRNA purification kit (Invitrogen) following manufacturers protocol. First strand cDNA was synthesized using two methods. The first method utilized a polyT-oligonucleotide with a GsuI restriction enzyme site incorporated into the sequence to result in removal of the poly-A tail. The second method utilized random hexamers (IDT, Coralville, IA). Second strand cDNA was synthesized followed by repair of the cDNA ends and addition of the Titanium 454 adapters following manufacturers protocol (Roche 454). Size selection of the product to isolate the 500–800 bp fragments was completed by nebulization of the sample (GsuI cDNA library) or the double Ampure bead (Beckman Coulter, Brea, CA) method (random hexamer cDNA library) following an abbreviated protocol (Roche 454). The size-selected fragment was normalized to remove overrepresented gene products using a duplex-specific nuclease (EVROGEN, Moscow, Russia) following manufacturers instructions. The resulting library was amplified and the SST (single strand template) library was isolated for analysis on the 454 sequencer.

### Emulsion Based PCR and Pyrosequencing

Emulsion based PCR (emPCR) was completed using the emPCR Kit (Roche 454) according to manufacturers procedures. A total of 10,054,000 beads were sequenced resulting in 2,324,269 reads that were delivered in FASTA format to the assembler. This key pass number (# of beads that yield sequence compared to the number of beads loaded) falls within the range recommended by Roche.

### Data Assembly and Analysis of Transcripts

The reads were assembled using the MIRA sequence analysis program [**20**]. Sequencing depth and coverage for the contigs were evaluated using the Hawkeye genome assembly viewer software [**46**]. All 121,071 unfiltered contigs from the MIRA analysis were repeat masked, then compared to the RefSeq mRNA and protein databases using BLASTN and BLASTX, respectively. The sequences that did not match were compared to the whole nucleotide (nr) database in an attempt to determine which sequences were likely to represent *bona fide* transcripts not previously annotated. The contigs that matched to RefSeq were mapped against Build 10 of the *Sus scrofa* genome using BLAT. The contigstats output file from MIRA was used to filter the contigs for those formed from at least 10 reads to reduce the contaminating artifacts of low complexity and low quality reads forming independent contigs of low depth.

### Identification of 3′ UTR Expressed in Porcine Skeletal Muscle

All 2,324,269 reads from 454 pyrosequencing of the two cDNA libraries were mapped against the 5,134 full length 3′ UTR for *Sus scrofus* represented in the 3′ UTR database (http://utrdb.ba.itb.cnr.it/) using Newbler (version 2.5.3) sequence analysis software (Roche 454). The full length *Sus scrofa* 3′ UTR were used as the reference sequence. The cDNA parameter was selected for the reference sequence. All remaining settings for Newbler were selected as default.

### Computational Identification of miRNA Target Sequence in the 3′ UTR

Identified 3′ UTR expressed in porcine skeletal muscle were evaluated for miRNA target sequences by the MiRanda and TargetScan analysis software [**22]**–[26]. The current databases for computational identification of miRNA-targeted sequences do not support porcine miRNA sequences. Therefore, for the MiRanda and TargetScan analysis human miRNA sequences were chosen to computationally identify genes targeted by the selected porcine miRNA (Table S1). The human miRNA sequence was compared to the porcine sequence and most sequences shared 100% nucleotide (nt) identity (Table S1). Difference in nt similarity for the remaining miRNA occurred at the 3′ end and did not alter the seed sequence located in the 5′ end of the miRNA sequence.

Target prediction for MiRanda and TargetScan compares the seed sequences for each miRNA family to the 3′ UTR sequences for genes of interest to predict miRNA targets using specific algorithms for each prediction program [**22–26;** Table S2; Table S3]. For this work the miRNA from Table S1 were used, after noting that the Sus and human miRNA sequence are identical in the seed area. The 3′ UTR sequences were obtained from assembled contigs of cDNA reads that mapped to the reference sequence where known 3′ UTR from the database also mapped. An additional requirement was that a RefSeq gene also mapped upstream and on the same strand and that cDNA contigs also mapped to the gene locus.

### KEGG Pathways for Expressed Transcripts

KEGG pathways for genes with 3′ UTR targeted by the selected porcine miRNA were determined using DB visualizer (http://www.dbvis.com) with the RefSeq match to its KEGG pathway through EntrezGene [**47;** Table S2; Table S3]. The EntrezGene pathway (PATH_ID and PATHWAY_DESCRIPTION) for each gene sequence is noted in Table S2 and Table S3.

## Supporting Information

Table S1 **MicroRNA sequence for highly abundant miRNA in porcine skeletal muscle with the associated human miRNA sequence.** MiRNA sequence for the highly abundant miRNA previously identified in porcine skeletal muscle (ssc) and corresponding human miRNA sequence (hsa).
(DOC)

Table S2 **3′ UTR of genes targeted by highly abundant miRNA in porcine skeletal muscle as predicted by MiRanda.** 3′ UTR expressed in porcine skeletal muscle (UTR_ID) were evaluated to computationally identify predicted miRNA binding sites by MiRanda (PREDICTED_miRNA_BINDING_SITES_MIRANDA). KEGG pathway (PATH_ID, PATHWAY_DESCRIPTION) was identified for each of the targeted genes (GENBANK ID, GENE_ID, GENE_SYMBOL) associated with the expressed 3′ UTR.
(XLS)

Table S3 **3′ UTR of genes targeted by highly abundant miRNA in porcine skeletal muscle as predicted by TargetScan.** 3′ UTR expressed in porcine skeletal muscle (UTR_ID) were evaluated to computationally identify predicted miRNA binding sites by TargetScan (PREDICTED_miRNA_BINDING_SITES_TARGETSCAN). KEGG pathway (PATH_ID, PATHWAY_DESCRIPTION) was identified for each of the targeted genes (GENBANK ID, GENE_ID, GENE_SYMBOL) associated with the expressed 3′ UTR.
(XLSX)

Table S4 **Overlap of 3′ UTR that contain predicted miRNA binding sites.** 3′ UTR expressed in porcine skeletal muscle were evaluated to computationally identify miRNA binding sites with MiRanda and TargetScan. Overlap of 3′ UTR (UTR_ID) between MIRANDA and TargetScan was determined.
(XLSX)
