# Supplementary material for: Next-Generation Sequencing of the Porcine Skeletal Muscle Transcriptome for Computational Prediction of MicroRNA Gene Targets
Source: PLoS One. 2012 Jul 27;7(7):e42039. doi: 10.1371/journal.pone.0042039 (PMC3407067; doi:10.1371/journal.pone.0042039)
Supplement: Table S1 — MicroRNA sequence for highly abundant miRNA in porcine skeletal muscle with the associated human miRNA sequence. MiRNA sequence for the highly abundant miRNA previously identified in porcine skeletal muscle (ssc) and corresponding human miRNA sequence (hsa). (DOC) [file pone.0042039.s001.doc]

**Table S1. MicroRNA sequence for highly abundant miRNA in porcine skeletal muscle with the associated human miRNA sequence.**

| **MicroRNA** | **Porcine MicroRNA Sequence** | **Human MicroRNA Sequence** |
| --- | --- | --- |
| miR-1 | ssc-UGGAAUGUAAAGAAGUAUGU | hsa-uggaauguaaagaaguauguau |
| miR-15a | ssc-UAGCAGCACAUAAUGGUUUGU | hsa-uagcagcacauaaugguuugug |
| miR-16 | ssc-UAGCAGCACGUAAAUAUUGG | hsa-UAGCAGCACGUAAAUAUUGGCG |
| miR-18b | ssc-UAAGGUGCAUCUAGUGCAGAUAG | hsa-UAAGGUGCAUCUAGUGCAGAUAG |
| miR-20a | ssc-UAAAGUGCUUAUAGUGCAGGUA | hsa-UAAAGUGCUUAUAGUGCAGGUAG |
| miR-22 | ssc-AAGCUGCCAGUUGAAGAACUGU | hsa-aagcugccaguugaagaacugu |
| miR-24 | ssc-UGGCUCAGUUCAGCAGGAACAG | hsa-uggcucaguucagcaggaacag |
| miR-26a | ssc-UUCAAGUAAUCCAGGAUAGGC | hsa-uucaaguaauccaggauaggcu |
| miR-27a | ssc-UUCACAGUGGCUAAGUUCUGC | hsa-uucacaguggcuaaguucugc |
| miR-29a | ssc-UAGCACCAUCUGAAAUCGGUUA | hsa-uagcaccaucugaaaucgguua |
| miR-34a | ssc-UGGCAGUGUCUUAGCUGGUUGU | hsa-UGGCAGUGUCUUAGCUGGUUGU |
| miR-106a | ssc-AAAAGUGCUUACAGUGCAGGUAGC | hsa-AAAAGUGCUUACAGUGCAGGUAG |
| miR-126 | ssc-UCGUACCGUGAGUAAUAAUGCG | hsa-UCGUACCGUGAGUAAUAAUGCG |
| miR-130a | ssc-CAGUGCAAUGUUAAAAGGGCAU | hsa-CAGUGCAAUGUUAAAAGGGCAU |
| miR-133a | ssc-UUUGGUCCCCUUCAACCAGCU | hsa-uuugguccccuucaaccagcug |
| miR-143 | ssc-UGAGAUGAAGCACUGUAGCUC | hsa-ugagaugaagcacuguagcuc |
| miR-148a | ssc-UCAGUGCACUACAGAACUUUGU | hsa-UCAGUGCACUACAGAACUUUGU |
| miR-151 | ssc-UCGAGGAGCUCACAGUCUAGU | hsa-UCGAGGAGCUCACAGUCUAGU |
| miR-185 | ssc-UGGAGAGAAAGGCAGUUCCUGA | hsa-UGGAGAGAAAGGCAGUUCCUGA |
| miR-199a-3p | ssc-ACAGUAGUCUGCACAUUGGUUA | hsa-ACAGUAGUCUGCACAUUGGUUA |
| miR-206 | ssc-UGGAAUGUAAGGAAGUGUGUGA | hsa-uggaauguaaggaagugugugg |
| miR-338 | ssc-UCCAGCAUCAGUGAUUUUGUUG | hsa-AACAAUAUCCUGGUGCUGAGUG |
| miR-376a | ssc-AUCAUAGAGGAAAAUCCAUGU | hsa-AUCAUAGAGGAAAAUCCAUGU |
| miR-381 | ssc-UAUACAAGGACAAGCUCUCUG | hsa-UAUACAAGGGCAAGCUCUCUGU |
| miR-423-5p | ssc-UGAGGGGCAGAGAGCGAGACUUU | hsa-UGAGGGGCAGAGAGCGAGACUUU |
| miR-424 | ssc-CAGCAGCAAUUCAUGUUUUGAA | hsa-CAGCAGCAAUUCAUGUUUUGAA |
| miR-432-5p | ssc-UCUUGGAGUAGGUCAUUGGGU | hsa-UCUUGGAGUAGGUCAUUGGGUGG |
| miR-450a | ssc-UUUUGCGAUGUGUUCCUAAUA | hsa-UUUUGCGAUGUGUUCCUAAUAU |
| miR-487b | ssc-AAUCGUACAGGGUCAUCCACUU | hsa-AAUCAUACAGGGACAUCCAGUU |
| miR-503 | ssc-UAGCAGCGGGAACAGUACUGCAG | hsa-UAGCAGCGGGAACAGUACUGCAG |
| miR-542-5p | ssc-UCGGGGAUCAUCAUGUCACGAGA | hsa-UCGGGGAUCAUCAUGUCACGAGA |
| miR-543 | ssc-AAACAUUCGCGGUGCACUUCUU | hsa-AAACAUUCGCGGUGCACUUCUU |
| miR-744 | ssc-UGCGGGGCUAGGGCUAACAGCA | hsa-UGCGGGGCUAGGGCUAACAGCA |
